# Molecular basis of RNA guanine-7 methyltransferase (RNMT) activation by RAM

**DOI:** 10.1093/nar/gkw637

**Published:** 2016-07-15

**Authors:** Dhaval Varshney, Alain-Pierre Petit, Juan A. Bueren-Calabuig, Chimed Jansen, Dan A. Fletcher, Mark Peggie, Simone Weidlich, Paul Scullion, Andrei V. Pisliakov, Victoria H. Cowling

**Affiliations:** 1 Centre for Gene Regulation and Expression, School of Life Sciences, University of Dundee, Dow Street, Dundee DD1 5EH, UK; 2 MRC Protein Phosphorylation and Ubiquitylation Unit, School of Life Sciences, University of Dundee, Dow Street, Dundee DD1 5EH, UK; 3 Drug Discovery Unit, Division of Biological Chemistry and Drug Discovery, School of Life Sciences, University of Dundee, Dow Street, Dundee DD1 5EH, UK; 4 Computational Biology, School of Life Sciences, University of Dundee, Dow Street, Dundee DD1 5EH, UK; 5 Physics, School of Science and Engineering, University of Dundee, Nethergate, Dundee DD1 5EH, UK; 6 Division of Signal Transduction Therapies, School of Life Sciences, University of Dundee, Dow Street, Dundee DD1 5EH, UK

## Abstract

Maturation and translation of mRNA in eukaryotes requires the addition of the 7-methylguanosine cap. In vertebrates, the cap methyltransferase, RNA guanine-7 methyltransferase (RNMT), has an activating subunit, RNMT-Activating Miniprotein (RAM). Here we report the first crystal structure of the human RNMT in complex with the activation domain of RAM. A relatively unstructured and negatively charged RAM binds to a positively charged surface groove on RNMT, distal to the active site. This results in stabilisation of a RNMT lobe structure which co-evolved with RAM and is required for RAM binding. Structure-guided mutagenesis and molecular dynamics simulations reveal that RAM stabilises the structure and positioning of the RNMT lobe and the adjacent α-helix hinge, resulting in optimal positioning of helix A which contacts substrates in the active site. Using biophysical and biochemical approaches, we observe that RAM increases the recruitment of the methyl donor, AdoMet (S-adenosyl methionine), to RNMT. Thus we report the mechanism by which RAM allosterically activates RNMT, allowing it to function as a molecular rheostat for mRNA cap methylation.

## INTRODUCTION

Eukaryotic mRNA is modified by the addition of the 5′ cap structure; 7-methylguanosine linked to the first transcribed nucleotide by a 5′-5′ triphosphate bridge (1,2). The cap marks RNA pol II transcripts for the unique series of processing events required for translation into protein (3,4). Complexes including the Cap-Binding Complex and Eukaryotic Initiation Factor 4F are recruited to the cap and mediate splicing, export and translation initiation. In addition, the cap protects transcripts from exonucleases during transcription, and the capping enzymes can promote transcriptional elongation (5,6). mRNA cap formation is upregulated by c-Myc oncogene leading to interest in the capping enzymes as therapeutic targets (7,8).

The mRNA cap is formed on the first transcribed nucleotide of transcripts by three sequential enzymatic activities; triphosphatase, guanylyltransferase and methyltransferase (4,9). The 5′ triphosphate of pre-mRNA is hydrolyzed to diphosphate by a 5′-triphosphatase, to which GMP is added by the RNA guanylyltransferase to create the cap intermediate, GpppN. The cap intermediate is methylated by the RNA guanine N-7 methyltransferase, utilising the methyl donor, AdoMet, to create the mature cap, m7GpppN, and byproduct, AdoHcy (S-adenosyl homocysteine). These activities have different configurations in different eukaryotic species and viruses, ranging from all being present on a single peptide to all being present on distinct peptides. In mammals, RNA guanylyltransferase and 5′-triphosphatase (RNGTT/CE) caps the nascent transcript and RNA guanine-7 methyltransferase (RNMT) methylates the cap.

RNA pol II transcripts are selectively capped because one or more of the capping enzymes is recruited to the large subunit C-terminal domain (CTD) when phosphorylated during the initial stages of transcription (4). The recruitment of eukaryotic capping enzymes to RNA pol II CTD has been extensively characterized, including elucidation of the mechanism by which the phosphorylated CTD activates the guanylyltransferase (2,10,11). Recent work in *Saccharomyces cerevisiae* demonstrated that the guanylyltransferase forms a stable complex with transcribing RNA pol II, with the active site facing the nascent transcript emerging from the RNA exit tunnel (12). Phosphorylated RNA pol II CTD also associates with the cap methyltransferase, although interaction with the human enzyme, RNMT, is probably indirect (2,13,14).

Much of our understanding of mRNA cap methylation comes from the structure of the microsporidian parasite, *Encephalitozoon cuniculi*, cap methyltransferase, Ecm1 (PDB: 1RI5) (15), and the Vaccinia virus enzyme D1 cap methyltransferase domain in complex with its activating subunit D12 (PDB: 2VDW) (16–18). Ecm1 and D1 contain similar Class I methyltransferase folds typical of the majority of methyltransferases, a conserved VLxI/ LxxGxGxDL motif and a deep AdoMet/ AdoHcy binding cleft. Structure–function analysis of superposed Ecm1 structures in complex with AdoMet, AdoHcy and the cap analogue m^7^GpppG revealed two ligand binding pockets which provide optimal positioning of substrates and a favourable electrostatic environment to catalyse the in-line transfer of the methyl group onto the N7 position of the guanosine cap (15,19). Similar ligand binding pockets were observed in the D1 methyltransferase (16,17).

Human RNMT is a 476 amino acid nuclear protein consisting of a catalytic domain (residues 121–476), with homology to other eukaryotic cap methyltransferases and an N-terminal regulatory domain (residues 1–120), that mediates recruitment to transcription initiation sites (13,14,20,21). The human RNMT structure was released by the Structural Genomics Consortium (SGC; PDB: 3BGV), and has considerable similarity to Ecm1 (RMSD: 1.25 Å) (22). RNMT also has an activating subunit, RAM (RNMT-activating miniprotein) (23). Although homologues of RNMT are present in all eukaryotes, RAM is only present in vertebrates leading to interest in its mechanism of action and cellular function.

RAM consists of an N-terminal RNMT activation domain (residues 1–55), a central RNA binding domain (residues 56–90), and a C-terminal PY nuclear localisation domain (residues 91–118) (23,24). The RAM RNA binding domain is required for cell viability but not for RNMT activation, and therefore may select specific critical transcripts for enhanced methylation. RAM 2–45 is the minimal fragment of RAM that binds to the catalytic domain of RNMT and enhances enzymatic activity; however, its mechanism of action is unclear. The Vaccinia virus D1 cap methyltransferase activating subunit, D12, increases the affinity of the enzyme for AdoMet and substrate, and increases *k*_cat_ (25). The structure of D1 cap methyltransferase in complex with D12 revealed extensive interactions between the two proteins and suggested an allosteric mechanism of activation (16,17). D12 is twice the length of RAM and appears unrelated in sequence and predicted structural motifs. Therefore, it is unclear whether D12 and RAM activate their methyltransferases by similar or distinct mechanisms.

In this study, we use X-ray crystallography to resolve the structure of the human RNMT-RAM complex. RAM binds to the RNMT surface distal to the active site, resulting in stabilisation of a series of RNMT structures including a lobe which co-evolved with RAM in vertebrates. RAM binding results in optimal orientation of key amino acids in the RNMT active site involved in substrate binding. Thus, we report the mechanism by which RAM and the RNMT lobe together form a molecular switch for mRNA cap methylation.

## MATERIALS AND METHODS

### Crystallisation conditions

RNMT 165–476 in complex with RAM 2–45 was crystallized at 17°C by sitting drop vapor diffusion method. Drops were made of 200 nl of the protein complex solution (34 mg/ml) mixed with 200 nl of the reservoir solution containing 0.1 M MES pH 6.5 and 30% PEG 4000. Crystals were flash-frozen in the reservoir solution mixed with 20% glycerol. RNMT 165–476 monomer was crystallized at 17°C by sitting drop vapor diffusion method. Drops were made of 200 nl of 35 mg/ml protein solution supplemented with thermolysin (1/5000 m/m) and 0.2 mM CaCl_2_ mixed with 200 nl of the reservoir solution containing 0.1 M MES pH 6.5 and 25% PEG 4000. Crystals were flash-frozen in the reservoir solution mixed with 25% glycerol. RNMT 165–476 Δ416–456 was crystallized at 4ºC using the hanging drop vapor diffusion technique. Drops were made of 1 μl of 10 mg/ml protein solution mixed with 1 μl of the reservoir solution composed by 0.1 M Hepes pH 7.8, 15% isopropanol and 6% PEG 4000. Crystals were flash-frozen in the reservoir solution mixed with 25% MPD.

### *In vitro* methyltransferase assay

Cap methyltransferase assays were performed according to Cowling, 2010 with minor alterations (26). In brief, specified concentrations of RNMT and RAM were incubated with an *in vitro* transcribed 55-nt ^32^P-capped RNA and 10 nM AdoMet for 5 min at 30°C, followed by 65°C for 20 min heat inactivation. RNA was digested with P1 nuclease and cap structures resolved on PEI cellulose in 0.4 M ammomium sulphate.

### GST-pulldowns

Two microgram recombinant GST or GST-RAM was incubated with equimolar His-RNMT and glutathione sepharose in salt wash buffer (50 mM Tris–Cl pH 7.5, 250 mM NaCl, 0.03% Brij-35, 1 mM DTT) at 4°C for 1 h. Resin was washed twice in 1 ml salt wash buffer, eluted with Laemmli buffer, resolved by SDS-PAGE and proteins stained with Coomassie blue.

### Fluorescence polarisation assay SAM-binding site probe

SAM-binding site probe (Cayman Chemical, Michigan, USA) was resuspended in 6 ml binding buffer (50 mM Tris–Cl pH 7.5, 6 mM KCl, 1.25 mM MgCl_2_, 0.01% Tween, 1 mM DTT), aliquotted and stored at −20°C. 1 μM final concentration of RNMT, RAM and BSA in binding buffer were loaded into 10 μl in 384-well low volume round bottom black assay plate (Corning). 1 μl 50 ng/μl capped RNA, 0.1 mM GpppG or dH_2_O plus 4 μl of SAM_FP_ probe was added as appropriate. Plate was incubated at 37°C for 30 min. Maximal polarisation (mP) measurements were made using the Texas Red FP mirror and filters on an EnVision^®^ 2104 multilabel plate reader (PerkinElmer). Data points were performed in triplicate in three independent experiments. 10 μm unlabelled SAM was added as a competitor. mP for probe binding to bovine serum albumin (BSA) was deducted as background.

### Immunoprecipitations and western blotting

Cells were lysed in 10 mM Tris–Cl pH 7.05, 50 mM NaCl, 50 mM NaF, 10% glycerol, 0.5% Triton X-100 containing protease inhibitors. 1–2 mg lysate were incubated with monoclonal anti-HA-agarose (Sigma-Aldrich) for 2 h at 4°C and washed four times with lysis buffer prior to elution by in Laemmli buffer, and western blot analysis. Sheep polyclonal antibodies used against full length RNMT and RAM were raised in house.

### Nuclear magnetic resonance (NMR)

NMR data was acquired on a Bruker AVANCE III HD 500 MHz NMR spectrometer equipped with a 5 mm QCI-F probe. The ^1^H–^15^N HSQC included suppression of the water by the WATERGATE pulse sequence (27,28). ^15^N-labeled RAM 2–45 protein was expressed in *Escherichia coli* BL21 Codon Plus RIL strain grown in minimal medium supplemented with ^15^NH_4_CL and purified as for X-ray crystallography at a concentration of 230 μM in 25 mM PIPES (pH 6.5, 175 mM NaCl, 25 mM KCl, 10% glycerol, 1 mM TCEP and 10% D2O). RMNT 165–476 (unlabeled)—RAM 2–45 (^15^N-labeled) complex was prepared as described for X-ray crystallography at a concentration of 160 μM in buffer conditions identical to monomeric RAM. The sample volume was 200 μl in a 3 mm NMR tube and temperature of the sample during acquisition was 25°C.

### Molecular dynamics (MD) simulations

Using the crystal structure of RNMT-RAM (PDB: 5E8J), six different simulation systems were constructed: (i) RNMT, (ii) RNMT–RAM, (iii) RNMT Δ419-458, (iv) RNMT R450E P452E, (v) RNMT W178C A417C and (vi) RNMT K409E K413E. For each system two sets of simulations were performed: (a) at a ‘standard’ temperature (300 K) for 400 ns and (b) at a ‘high’ temperature (400 K) for 60 ns. In addition, a 50 ns MD simulation (at 300 K) was carried out for the RNMT–RAM complex with the ligands (AdoMet and Gppp) bound in the active site. All MD simulations were performed in the AMBER14 package (29) using the ff14SB force field (30). Supplementary Table S2 provides the summary of the MD simulations performed.

See supplementary methods for details of recombinant protein purification, X-ray diffraction data collection, mass spectrometry, cell culture and MD simulations (including supplementary references 37–53).

## RESULTS

### Structure of the human RNMT-RAM complex

**Figure 1.**
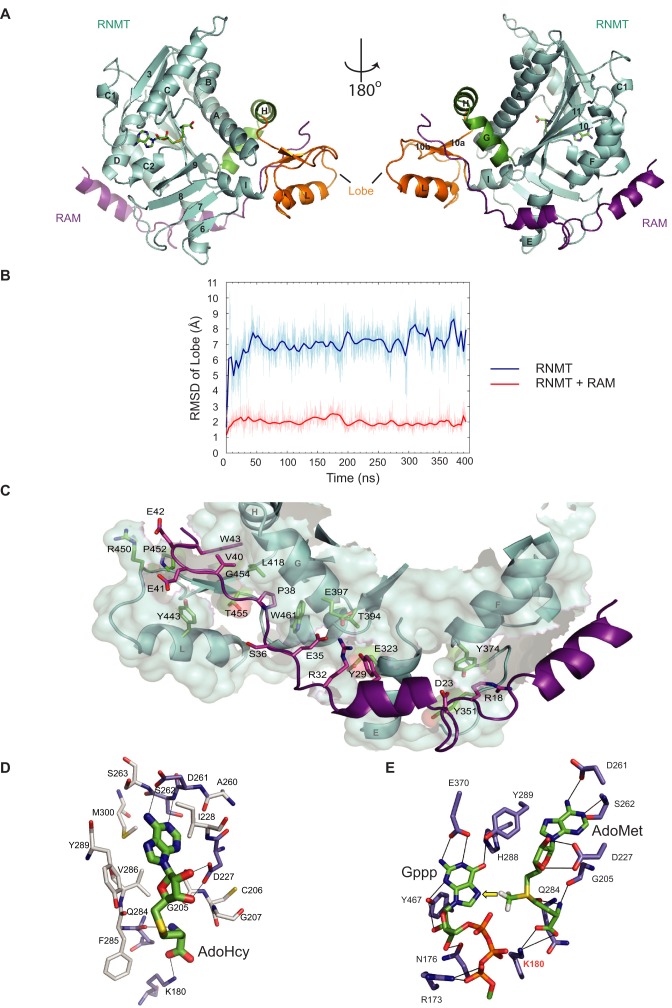
RNMT-RAM crystal structure. (**A**) RNMT 165–476 (cyan) and RAM 2–45 (magenta) crystallised with AdoHcy. The RNMT lobe is highlighted in orange and α-helix hinge in green. Secondary structure attribution was performed with DSSP. (**B**) Time evolution of RMSD of the lobe backbone (residues 419–458) in molecular dynamics simulations, with RAM (red) and without (blue). The RNMT-RAM crystal structure was used as a reference. (**C**) RNMT and RAM amino acids involved in polar interactions as determined using the PISA server (EMBLI-EBI) are shown as sticks. (**D**) AdoHcy binding site in RNMT shown with amino acids involved in hydrogen bond formation (purple) and hydrophobic interactions (white). (**E**) Representative structure from MD simulation of RNMT–RAM with the ligands AdoMet and Gppp. Initial positioning of Gppp was based on previous structure (PDB: 1RI2) (15). For clarity, only residues involved in electrostatic interactions are shown. The yellow arrow depicts in-line transfer of the methyl group. Lines represent hydrogen bonds in panels D and E.

Here we report the structure of human RNMT in complex with RAM. The RNMT catalytic domain (residues 165–476) was crystallised in complex with the RNMT activation domain of RAM (residues 2–45) and the methylation byproduct, AdoHcy (S-adenosyl homocysteine), and solved at a resolution of 2.3 Å (PDB: 5E8J; Figure 1A and Table 1). Two complexes within an asymmetric unit were found to perfectly superpose (RMSD 0.46 Å RNMT chain A versus B; RMSD 0.22 Å RAM chain C versus D). The structure of RNMT within RNMT–RAM is not substantially different from that of the RNMT monomer previously determined by the Structural Genomics Consortium (RMSD 0.62 Å PDB: 5E8J versus PDB: 3BGV). The presence of RAM does not alter the canonical Class I methyltransferase fold in RNMT, however it allows the refinement of a lobe structure (residues 416–456), which was absent in 3BGV. Secondary structure element attribution using the DSSP program revealed the lobe to consist of a β-sheet made of two small anti-parallel β-strands (10a and 10b), which flank the α-helix L (Figure 1A). Crystals were also obtained with the RNMT 165–476 monomer using limited in-drop proteolysis (PDB: 5E9W; Supplementary Figure S1A and Table 1). Whilst high quality diffraction data (2.3 Å) was obtained, the lack of density in the lobe region indicated that the lobe was absent in the protein crystals. Moreover, only low quality crystals of the RNMT 165–476 monomer could be obtained using sparse matrix screens without proteolysis. Thus, binding of RAM permits stabilisation of a lobe structure, RNMT 416–456. This was supported by molecular dynamics (MD) simulations that were used to obtain information on the kinetic and thermodynamic properties of RNMT, in the absence or presence of RAM (31). In the absence of RAM, the lobe region is highly disordered in the simulations, as reflected by its relatively high RMSD of 7.0 Å (Figure 1B and Supplementary Movie 1). RMSD values in MD simulations provide a measure of how much the structure changes during a simulation, and reflect the degree of conformational change and flexibility in the system. RAM binding greatly stabilizes the lobe (RMSD 2.0 Å), through multiple RNMT–RAM interactions, most of which are preserved from the crystal structure (see later).

**Table 1.** Crystallographic model refinement and data collection statistics

Sample	RNMT 165–476 - RAM 2–45	RNMT 165–476 limited proteolysis	RNMT 165–476 Δ416-456
PDB code	5E8J	5E9W	5E9J
**Data measurement**			
Source	DLS ID24	DLS ID04	ESRF ID30
Space group	*P*1	*P*2_1_2_1_2_1_	*P*2_1_2_1_2_1_
Unit cell (Å/°)	*a* = 49.15, *b* = 50.49, *c* = 84.58, *α* = 90.06, *β* = 92.41, *γ* = 115.41	*a* = 77.46, *b* = 99.16, *c* = 167.61, *α* = *β* = *γ* = 90.00	*a* = 70.76, *b* = 114.38, *c* = 134.81, *α* = *β* = *γ* = 90.00
Resolution (Å)	29.44–2.35 (2.48–2.35)	29.58–2.28 (2.41–2.28)	87.21–3.47 (3.71–3.47)
Observations	45164 (5607)	160886 (23186)	67860 (6796)
Unique observations	28355 (3888)	57298 (8296)	13618 (1770)
Rmerge(%)	0.11 (0.49)	0.08 (0.27)	0.21 (0.97)
*I*/*σI*	6.6 (2.0)	10.3 (3.9)	6.6 (1.6)
Completeness (%)	92.8 (87.1)	97.0 (97.6)	92.2 (67.6)
Multiplicity	1.6 (1.4)	2.8 (2.8)	4.9 (3.8)
**Refinement Statistics**			
Resolution range (Å)	28.16–2.35	29.57–2.28	48.80–3.47
*R*-factor *R*_work_/*R*_free_	0.21/0.26 (0.26/0.32)	0.22/0.25 (0.27/0.32)	0.23/0.26 (0.33/0.40)
Molprobity score	2.15	1.82	2.41
Number of atoms*			
Protein	5807	8989	4441
Ligand	96	104	90
Water	219	307	0
Mean *B*-factor (Å^2^)	24.55	22.42	91.00
RMS bond length deviation (Å)	0.003	0.008	0.003
RMS angle deviation (°)	0.790	1.342	0.784
Residues in favored region of the Ramachandran plot (%)	95.7	95.9	94.8
Residues in allowed region of the Ramachandran plot (%)	3.3	3.3	4.9

*Hydrogen atoms are not taken into account.

Values in parentheses are for the outer shell.

### The structure of RNMT-RAM and Ecm1 are comparable

**Figure 2.**
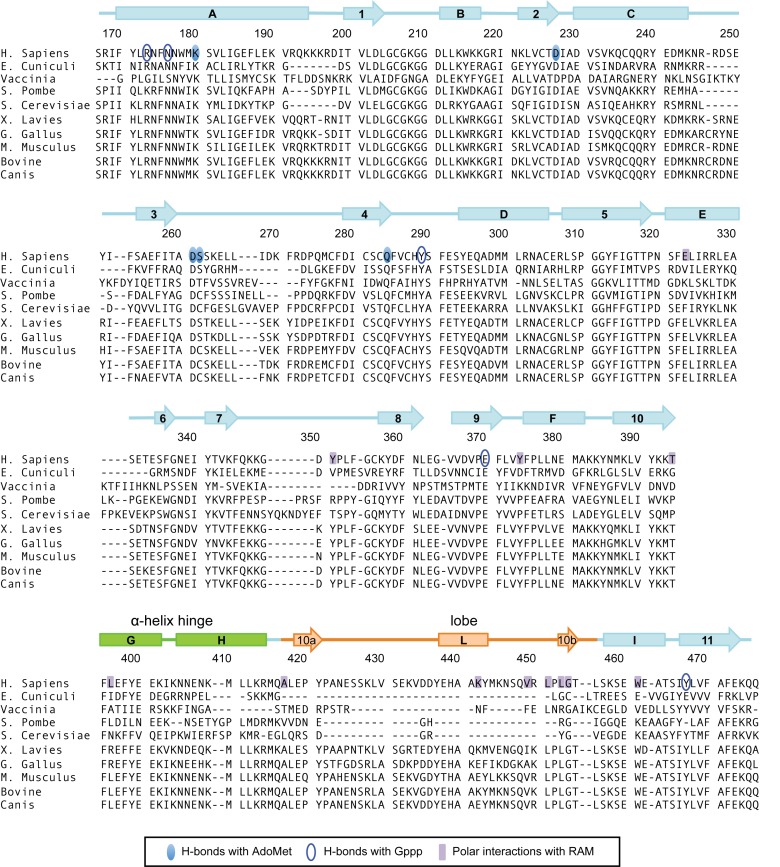
Sequence alignments and secondary structure element attribution for human RNMT. Amino acid sequence alignment using EMBL-EBI Clustal Omega between RNMT orthologs from a selection of eukaryotic organisms. Secondary structure attribution for RNMT (PDB: 5E8J) was performed with DSSP. The nomenclature of structural features shown has been transcribed from Fabrega *et al*. (15). Previously unidentified features include strands 10a, 10b and helix L, which lies within the lobe structure (orange) found to be conserved amongst vertebrates. Helices H and G form the α-helix hinge (green). Amino acids involved in polar interaction with AdoMet, Gppp and RAM are highlighted.

We compared the RNMT crystal structure to that of *E. cuniculi* mRNA cap methyltransferase, Ecm1, which has been analysed previously (15). Despite having <40% sequence identity, human RNMT in complex with RAM has considerable structural similarity to Ecm1 (RMSD 1.31 Å PDB: 5E8J versus PDB: 1RI3; Supplementary Figure S1B). The sequence alignment in Figure 2 depicts key regions of homology and functional residues of a selection of mRNA cap methyltransferases. The only major difference between the Ecm1 and RNMT crystal structures is the presence of the RNMT lobe, residues 416–456, which is absent in Ecm1 (Figure 2). Of note, the lobe is also absent in the cap methyltransferases of *S. cerevisae* (ABD1), *S. pombe* (Pcm1) and Vaccinia virus D1. Minor structural differences between RNMT and Ecm1 include different length of helix A (RNMT residues 170–194, Ecm1 residues 44–62), and β-strand 9 (RNMT residues 365–371, Ecm1 residues 223–226), and two loops that do not superpose (RNMT residues 245–253 and 346–356). These differences are not due to RAM binding, since the RNMT monomer and RNMT–RAM structures superpose with RMSD of 0.5 Å (Supplementary Figure S1A).

### The structure of the RAM RNMT activation domain

The RNMT activation domain of RAM (residues 2–45) comprises of two α-helices (residues 4–14 and 24–31) and a region (residues 32–45) to which no fold was assigned (Figure 1c). We used NMR spectrometry to determine the structure of monomeric RAM 2–45 in solution (Supplementary Figure S1C). The HSQC of the unbound RAM shows that the majority of backbone amide peaks lie within a narrow range of chemical shifts in the proton dimension (7.9-8.5 PPM). There are 3–5 peaks at lower ^1^H chemical shifts which may indicate some small region(s) of ordered structure. However, the signals broadened significantly in presence of RNMT, showing a wider range of chemical shifts. These data indicate that RAM 2–45 is likely to be flexible in solution, but stabilised by binding to RNMT.

In the RNMT–RAM crystal structure, RAM 2–45 binds to a positively charged surface groove on RNMT, distal to the active site (Figure 1a, c and Supplementary Figure S2A). This groove comprises of the RNMT α-helix hinge containing helices G and H (residues 395–415) and the lobe. Helices G, H and I were previously described as unique to RNA methyltransferases (15). The interface between RNMT and RAM was analysed using the PISA server (EMBLI-EBI) revealing an average interface area of 1695 Å^2^. All interactions were identical in the two complexes of the asymmetric unit. Eleven amino acids in RAM spanning R18 to W43 are involved in polar interactions with RNMT (Supplementary Table S1), and RAM E25, R32 and E42 establish salt bridges with RNMT K392, E397 and R450. Further residues involved in hydrophobic interactions (within 4 Å) include RAM A7, V8, F11, F15, F19, Y26, Y29, P37, P38, I39, V40 and W43 and RNMT Y294, F322, I325, Y351, L353, L372, Y374, P376, L377, M381, Y399, L412, L418, P420, Y443 and W461. Many residues involved in these interactions lie in the RNMT lobe (residues 416–456), which co-evolved with RAM in vertebrates (Figure 2). All interactions between RNMT and RAM, remain unchanged throughout MD simulation, reflecting the stable interaction of these proteins (Supplementary Table S1).

### The structure of the RNMT active site

Crystal structures did not indicate significant differences in the interaction of AdoHcy with RNMT-RAM and Ecm1. The five RNMT amino acids involved in hydrogen bonding with AdoHcy (K180, G205, D227, D261, S262) are conserved in Ecm1, and perfectly superposed between the structures (Figure 1D and Supplementary Figure S2B). Since significant structural alterations were not observed in the active site, the binding poses of the ligands in the Ecm1 structure (PDB: 1RI2) were adopted as a starting point for modelling. The binding interactions were modelled and optimized in a 50 ns MD simulation. The equilibrated structure of the active site reveals that Gppp interacts with RNMT via a network of hydrogen bonds conserved from Ecm1 (Figure 1E). Throughout MD simulations, the sulphur centre (S*D*) and C*E* of AdoMet and the N*7* of Gppp display a nearly linear orientation (angle ∼160º), maintaining a distance of about 3.6 Å between N*7* (Gppp) and C*E* (AdoMet), thus providing an ideal configuration of reactants for in-line methyl transfer (Supplementary Figure S2C). Modelling predicts that the cap triphosphate bridge establishes hydrogen bonds with R173. The interaction of K180 in helix A with the carboxylate group of AdoMet remains stable in MD simulations, but in addition K180 also remains proximal to the α-phosphate of the cap (Figure 1E and Supplementary Figure S2D). K180 is likely to be critical for the ligand orientation required for optimal methyl transfer. Mutation of the equivalent residue in Ecm1 (K54) abolishes activity (15).

### The RNMT lobe is required for RAM binding and activity

**Figure 3.**
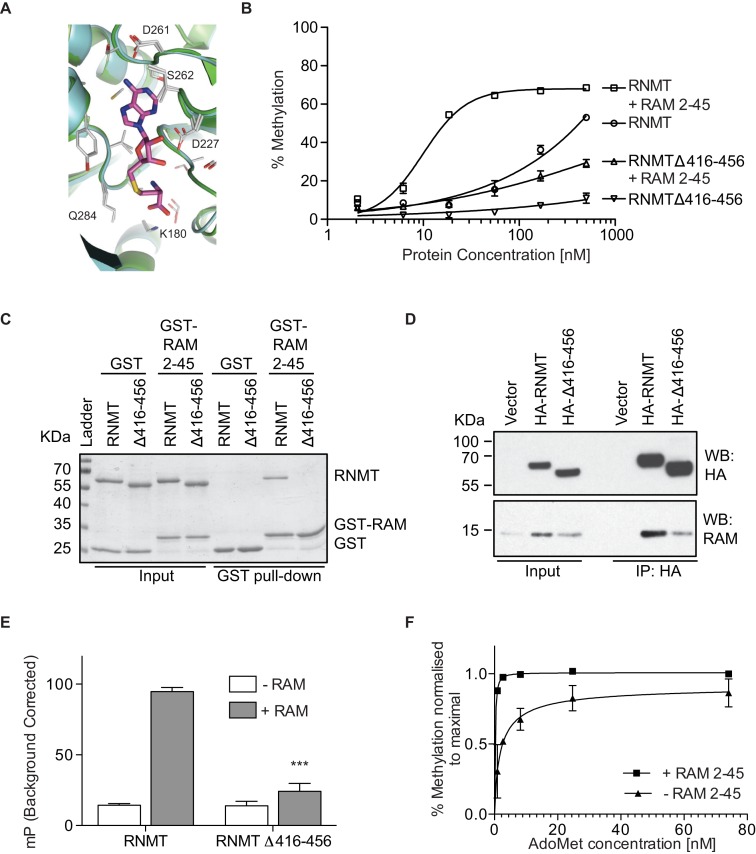
RAM increases AdoMet recruitment by RNMT. (**A**) Alignment of residues involved in AdoHcy binding for RNMT 165–476–RAM 2–45 (cyan) and RNMT 165–476 Δ416–456 monomer (green). Residues from both structures involved in polar interactions are shown as sticks. (**B**) Methyltransferase activity assays performed with titrations of RNMT and RNMT Δ416-456 in presence or absence of RAM (*n* = 3). (**C**) GST-pulldown of RNMT and RNMT Δ416–456 with GST-RAM 2–45. GST provides the negative control. (**D**) Co-immunoprecipitation followed by Western blot analysis on lysates from cells transiently transfected with vector only, HA-RNMT and HA-RNMT Δ416–456. (**E**) Maximal polarisation for SAM_FP_ probe binding to RNMT and RNMT Δ416-456 in absence or presence of RAM 2–45. 0.1 mM GpppG present throughout (*n* = 3). *P*-values are relative to RNMT controls where *** represents *P* < 0.001. (**F**) Methyltransferase activity assays performed with 100 nM RNMT in presence or absence of equimolar RAM 2–45 and an AdoMet titration (*n* = 2). Values in B, E and F represent mean ± S.D. Panels C and D represent two or more independent experiments.

The most overt effect of RAM binding is the stabilisation and resolution of the RNMT lobe, residues 416–456. In order to characterise RNMT lobe function, a deletion mutant was engineered in which lobe residues 416–456 are replaced with a GSGG linker (RNMTΔ416–456). The catalytic domain of the resultant RNMT monomer was crystallized (PDB: 5E9J; Table 1). Despite the absence of the lobe in this mutant and in thermolysin-cleaved RNMT, the crystal structures are not altered significantly compared to RNMT–RAM (RMSD 0.5 Å; Supplementary Figure S1A). Moreover, no substantial differences in the position of key active site residues were observed in the crystals upon deletion of the lobe (Figure 3A).

The activity of full-length RNMT and the lobe deletion mutant, RNMTΔ416–456, was compared. Removal of the lobe significantly reduced cap methyltransferase activity, over a titration of RNMT, with or without equimolar RAM 2–45, indicating that the lobe has a significant role in catalysis (Figure 3B). We have previously reported that an equimolar RAM to RNMT ratio is required for maximal enzymatic activation (23). Since RAM can partially activate RNMTΔ416–456, it activates by mechanisms additional to stabilising the lobe (discussed later). Many lobe residues interact with RAM and deletion of the lobe considerably weakened the RNMT-RAM interaction to below the limit of detection in GST-pulldown (Figure 3c). Furthermore, when expressed in HeLa cells, RNMTΔ416–456 bound weakly to endogenous RAM (Figure 3D). The IP/Input ratios calculated by image densitometry show a 2-fold reduction in binding efficiency for the mutant (IP/Input: 1.2) when compared to the wild-type protein (IP/Input: 2.4). Since RNMT and RAM expression is co-dependent, the lobe mutant was unable to elevate RAM expression equivalently to wild-type RNMT. In summary, deletion of the lobe reduces the catalytic activity of RNMT and impairs its ability to bind and be activated by RAM.

### The RNMT lobe and RAM promote AdoMet recruitment

Although RAM binding does not appreciably alter the RNMT active site observed in the crystal structure, it was important to determine if it alters ligand binding in solution. We evaluated AdoMet binding to RNMT using a fluorescent AdoMet-analogue, SAM_FP_ (Cayman Chemical), which was incubated with RNMT and its interaction detected as fluorescence polarisation (Supplementary Figure S3A). Increased polarisation from SAM_FP_ binding to RNMT-RAM was competed by excess AdoMet, validating the interaction of the probe with the AdoMet binding pocket of RNMT. This assay therefore enables the direct measurement of AdoMet binding by RNMT. The presence of GpppG or GpppG-RNA (guanosine-capped transcript) enhanced SAM_FP_ binding to RNMT-RAM, indicating a co-operative or ordered binding model (Supplementary Figure S3B). Co-operative binding of GpppG and AdoMet was also previously suggested for the Vaccinia virus cap methyltransferase (32). RAM 2–45 monomer did not exhibit SAM_FP_ binding, however it elevated SAM_FP_ binding to RNMT over 6-fold (Figure 3E and Supplementary Figure S3B). Deletion of the lobe did not impact on SAM_FP_ binding to the RNMT monomer, however RAM 2–45 was unable to stimulate SAM_FP_ binding to RNMTΔ416–456, consistent with a defect in RAM binding (Figure 3E). Data presented thus far indicates that RAM promotes AdoMet recruitment. A prediction from these data is that RAM should reduce the dependency of RNMT on AdoMet. Indeed, when cap methyltransferase assays were performed over a titration of AdoMet, RAM increased cap methyltransferase activity at low AdoMet concentrations (Figure 3F).

**Figure 4.**
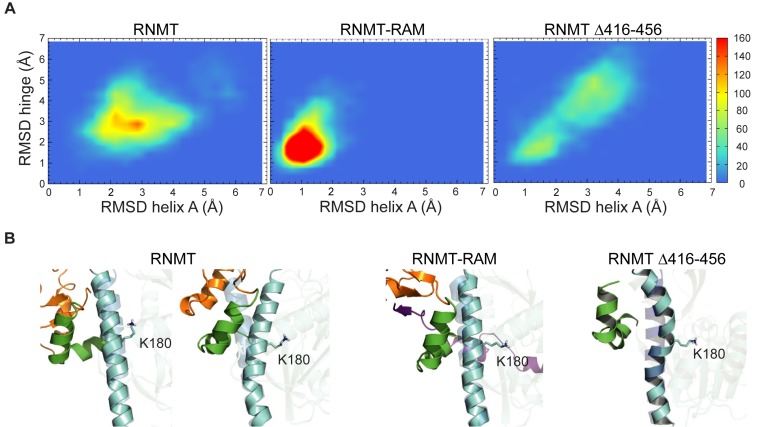
RAM stabilises RNMT in molecular dynamic simulations. (**A**) Heatmaps displaying backbone RMSD of the hinge (residues 395–416) versus RMSD of helix A (residues 341–345), calculated from 60ns high temperature MD trajectories of RNMT, RNMT–RAM and RNMTΔ416–456. The colour scale represents the number of snapshots in each ‘bin’. (**B**) Representative snapshots corresponding to the most sampled configuration in panel A. Helix A is shown in blue, hinge in green and lobe in orange. High-transparency image of helix A in the background represents the position in RNMT–RAM crystal structure.

MD simulations were employed to model the effect of RAM binding on the internal dynamics of different regions of RNMT. In simulations, RAM provided stability to the α-helix hinge and helix A of RNMT as visualized by RMSD values of these regions (Figure 4A). Three hydrogen bonds allow the interaction of the hinge with helix A (W178-N408, E186-K402 and N174-Q416), in an interface that also contains multiple hydrophobic residues. Helix A runs adjacent to the active site and includes K180, which interacts with AdoMet. The α-helix hinge and helix A keep their original positions in the RNMT–RAM complex in high temperature simulations, whilst in the absence of RAM they take a range of different conformations, including some with a severely distorted/kinked helix A (Figure 4B and Supplementary Movie 1). Removal of the lobe in RNMTΔ416–456 leads to a significant increase in flexibility of both the α-helix hinge and helix A in simulations. In agreement, the average isotropic B factor values for the α-helix hinge compared to the whole protein within the same crystal structure displays a marked increase on lobe removal (average *B* factor hinge = 28.2 Å^2^ versus main chain = 23 Å^2^), in comparison to the RNMT-RAM complex (average *B* factor hinge = 24.4 Å^2^ versus main chain = 24.4 Å^2^). The increased flexibility of the α-helix hinge therefore correlates with the loss of activity in the absence of RAM or following lobe deletion (Figure 3B).

### Mutations that stabilise the RNMT α-helix hinge reduce RAM dependency

**Figure 5.**
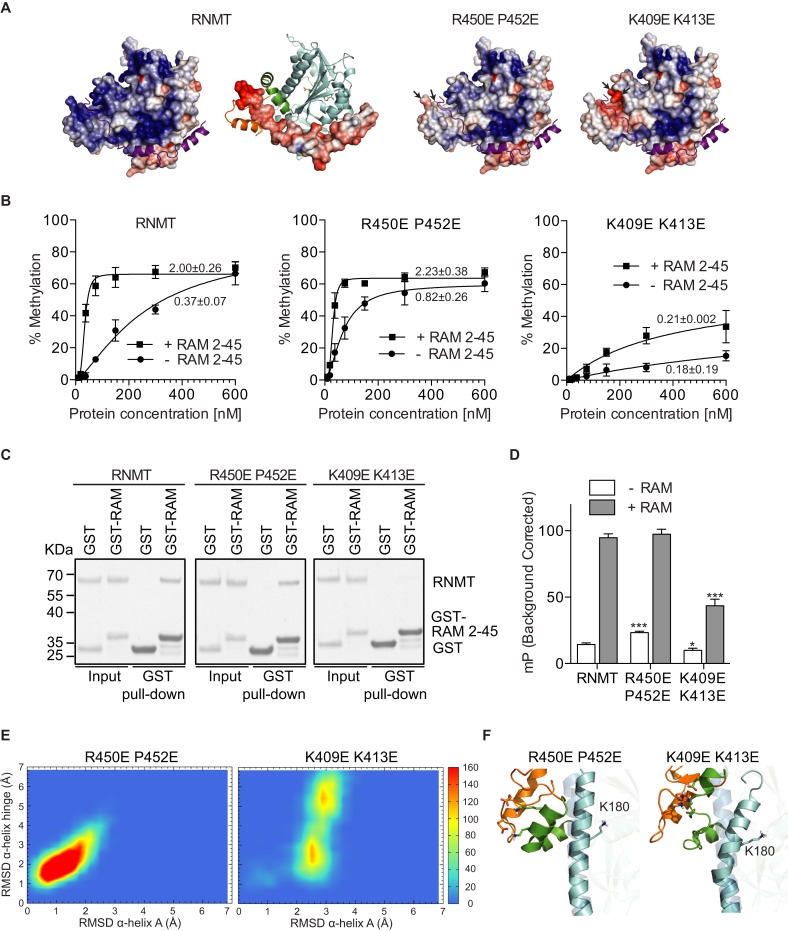
Reversing RNMT lobe charge increases enzymatic activity. (**A**) Electrostatic potential as calculated by APBS from the crystal structure of RNMT-RAM. Positive charge is depicted in blue and negative charge in red (–5/+5 KbT/e). Predicted alterations in charge due to amino acid substitutions on the lobe surface (P452E R450E) or surface facing the lobe (K409E K413E) are demonstrated. (**B**) Methyltransferase activity assays performed on RNMT, RNMT R450E P452E and RNMT K409E K413E in absence or presence of RAM (*n* = 3). Activity was calculated as % methylation per ng RNMT in a 5-minute reaction. Maximal activity over the titration is reported. (**C**) GST-pulldown of RNMT, RNMT R450E P452E and RNMT K409E K413E with GST-RAM 2–45. GST provides the negative control. (**D**) Maximal polarization for SAM_FP_ probe binding to RNMT, RNMT P450E R452E and RNMT K409E K413E in absence or presence of RAM 2–45. (**E**) Heatmaps displaying backbone RMSD of the hinge (residues 395–416) versus RMSD of helix A (residues 341–345) as calculated from 60ns high-temperature MD trajectories of RNMT R452E R450E and RNMT K409E K413E mutants. The color scale represents the number of snapshots in each ‘bin’. (**F**) Representative snapshots corresponding to the most sampled configuration in panel E. Helix A is shown in blue, hinge in green and lobe in orange. High-transparency image of helix A in the background represents the position in RNMT–RAM crystal structure. Values in panels B and D represent mean ± SD (*n* = 3). *P*-values in panel D are relative to RNMT controls where * represents *P* < 0.05 and *** represents *P* < 0.001.

The interaction of RAM and RNMT is largely charge-based, in particular the electrostatic interaction between the negatively charged RAM residues 35–45 and the positively charged groove between the lobe and the α-helix hinge of RNMT (Figure 5A and Supplementary Figure S2A). In order to investigate whether RAM functions to neutralise and stabilise repulsive forces between the RNMT lobe and α-helix hinge, charge-altering amino acid substitutions, R450E P452E, were made on the lobe (Figure 5A). These substitutions were predicted to reduce the requirement for charge stabilisation by RAM. Consistent with this hypothesis, the RNMT R450E P452E monomer exhibited a two-fold increase in activity compared to the wild-type RNMT monomer (Figure 5B). RNMT R450E P452E retained interaction with RAM 2–45 (Figure 5C), which increased activity only to the level observed for RNMT-RAM (Figure 5B). These data have been confirmed with a more extensive mutant RNMT R450E L451E P452E (Supplementary Figure S4A). The increased basal activity of RNMT R450E P452E is consistent with increased SAM_FP_ binding (Figure 5D). MD simulations of RNMT R450E P452E indicate altered interactions in the α-helix hinge region and significant structural rearrangements of the modified lobe (Figure 5E; Supplementary Movie 2). Early in the simulation, the lobe approaches the α-helix hinge and as a result K409 and K413 form salt bridges with R450E and P452E, respectively, mimicking equivalent interactions observed in the RAM–RNMT complex (Figure 5F, Supplementary Figure S4B and Supplementary Movie 2). Thus, the immediate effect of the modified lobe is a stabilization of the hinge, helix A and the nearby active site residues, as previously observed for the RNMT–RAM complex.

### Amino acids substitutions which disrupt or constrain the hinge reduce RNMT catalytic activity

In order to further probe the function of the α-helix hinge, the RNMT K409E K413E mutant was created, with the aim of disrupting the hinge surface charge distribution (Figure 5A). RNMT K409E K413E monomer exhibited reduced catalytic activity and reduced SAM_FP_ binding (Figure 5B and C). Furthermore, RNMT K409E K413E has a reduced interaction with RAM and reduced RAM-dependent stimulation of SAM_FP_ binding and activity (Figure 5B–D). In MD simulations the K409E K413E mutation resulted in distortion of the α-helix hinge together with helix A, which is kinked and tilts away from the hinge (Figure 5E). Importantly, the side chain of K180 is forced away from its original location to adopt a new position where it directly competes with the charged amino group of AdoMet (Figure 5F and Supplementary Figure S4B). This explains a significant reduction of SAM_FP_ binding to RNMT K409E K413E. The RNMT K409E K413E R414E mutant exhibited a similar loss in activity (Supplementary Figure S4A). These findings demonstrate that the α-helix hinge of RNMT is critical for AdoMet binding, RAM binding and catalytic activity.

**Figure 6.**
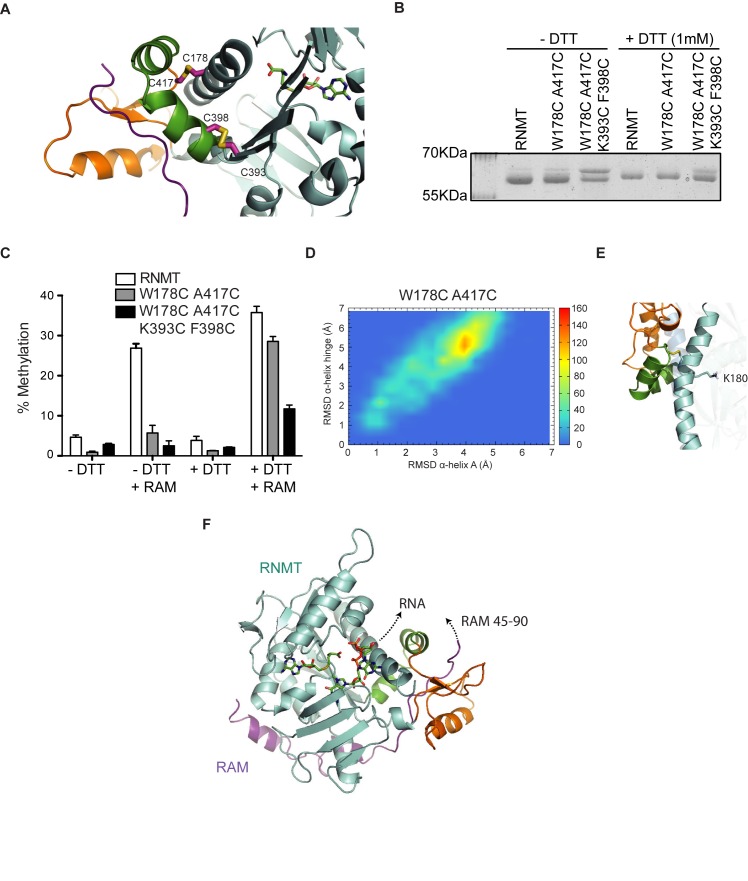
Restraining the RNMT α-helix hinge ablates its catalytic activity. (**A**) Predicted structures for amino acid substitutions introducing disulphide bridges designed to constrain the alpha helix hinge (green) to helix A. Introduced disulphide bridges are shown as sticks. (**B**) Coomassie stained SDS PAGE gels resolving RNMT, RNMT W178C A417C (single-bridge mutant) and RNMT W178C A417C K393C F398C (double-bridge mutant) in the absence and presence of 1 mM DTT. (**C**) Methyltransferase activity assays performed on 100nM RNMT, RNMT W178C A417C and RNMT W178C A417C K393C F398C, with or without RAM 2–45 with or without 1mM DTT. (**D**) Heatmaps displaying the backbone RMSD of the hinge (residues 395–416) versus RMSD of helix A (residues 341–345) as calculated from the 60ns high-temperature MD trajectories of RNMT W178C A417C. The color scale represents the number of snapshots in each ‘bin’. (**E**) Representative snapshots corresponding to the most sampled configuration in panel D. Helix A is shown in blue, hinge in green and lobe in orange. High-transparency image of helix A in the background represents the position in RNMT–RAM crystal structure. (**F**) MD modelling of the first nucleotide binding mode on the model of best fit obtained from densities by Fabrega *et al*. (15) (PDB: 1RI2). The predicted direction of the RNA and RAM RNA binding domain (residues 45–90) are indicated. Values in panels C and D represent mean ± S.D. (*n* = 3).

The previous mutants indicated that RAM draws the α-helix hinge towards the RNMT lobe thus aligning adjacent helix A, including K180, optimally for AdoMet binding. In order to create a mutant in which helix A is experimentally prevented from correct positioning, two or four substitutions were made in RNMT (W178C A417C or W178C A417C K393C F398C) with the aim of creating one or two disulphide bridges between the α-helix hinge and helix A (Figure 6A). Formation of disulphide bridges could be visualised by reduced RNMT mobility in SDS-PAGE, which could be partially rescued by reducing agent (Figure 6B). Both the single-bridge RNMT (W178C A417C) and the double-bridge RNMT (W178C A417C K393C F398C) mutants were defective for enzymatic activity in the absence or presence of RAM (Figure 6C). However, reducing disulphide bridges with DTT almost entirely restored the ability of RAM to activate the single-bridge mutant. The double-bridge mutant could only be partially activated by RAM upon the addition of reducing agent; however, conditions were insufficient to cleave the disulphide bridges completely, as evident by a double band remaining in SDS-PAGE. It must be noted that the addition of DTT did increase the activity of the wild-type RNMT-RAM complex marginally (1.3-fold), however its effects were greater for the single-bridge mutant (5-fold). In MD simulation of the single-bridge RNMT mutations, as designed the α-helix hinge was displaced due to the presence of disulphide bond. This affected the conformation of helix A and displaced the side chain of K180 to a position where it competes with AdoMet for its binding site (Figure 6d, e and Supplementary Figure S4B). Taken together, these data support the model that RAM draws the α-helix hinge towards the lobe, positioning helix A and K180 for optimal interaction with AdoMet.

### RNA exiting the RNMT active site is predicted to contact the RAM RNA-binding domain

Since the α-helix hinge lies adjacent to the RNA exit site, it may influence the ability of RNMT to bind to RNA. However, the currently available model (PDB: 1RI2) has m7Gppp built but lacks densities for the first nucleotide (15). The m7Gppp densities in 1RI2 were used to build a m7Gppp model within the active site of RNMT, and the model was optimized in the initial MD simulation. The first nucleotide guanine was then built manually onto the ligand coordinates and a second round of MD optimisation was performed to predict the binding mode for the first nucleotide. This revealed that the m7GpppG structure is bent, consistent with the previously observed model (Figure 6F). The first nucleotide is predicted to be exposed to solvent and consequently would be too agitated to be observed in density maps. Using the orientation of the O3′group of the first nucleotide, we can predict that RNA exiting the active site would interact with the central RNA binding domain of RAM (residues 45–90). Moreover, the optimal positioning of the α-helix hinge would contribute to the ability of RNMT to recruit RNA substrates.

## DISCUSSION

The eukaryotic mRNA cap methyltransferases have conserved biochemical and cellular functions. However, the RNMT activator RAM is only found vertebrates, which raises the question of why RAM evolved (23,33). The RNMT–RAM crystal structure in conjunction with biochemical analyses and molecular dynamics simulations has revealed that RAM activates RNMT by stabilising the RNMT lobe and α-helix hinge, thus positioning adjacent helix A in the active site in a position favourable for substrate binding and the methylation reaction.

### RNMT lobe, α-helix hinge and helix A govern activity

RAM binds distal to the RNMT active site and does not alter its crystal structure substantially when compared to the RNMT monomer or the *E. cuniculi* methyltransferase Ecm1 (15). RAM binding, however, stabilises the RNMT lobe (residues 416–456), a domain missing from structures of RNMT monomers. Analysis of cap methyltransferases across species indicates that the RNMT lobe co-evolved with RAM in vertebrates as a cap methyltransferase activation module. In molecular dynamics simulations, the lobe is highly flexible in the RNMT monomer, but stabilised by extensive interactions with RAM residues 36–42.

Stabilisation of the RNMT lobe by RAM impacts on AdoMet binding and catalytic activity as a result of a series of interactions, leading to changes in the dynamics of the active site. These changes, however, are not apparent in the crystal structures. Thus, we used MD simulations to explain observed alterations in catalytic activity following structure-guided amino acid substitutions. The adjacent surfaces of the α-helix hinge and lobe are positively charged and therefore, given their proximity, repulsive electrostatic forces destabilise these two elements in the RNMT monomer. This can be visualised in simulations in which in the absence of RAM both the α-helix hinge and lobe exhibit disorder and a higher degree of movement. The RNMT R450E P452E mutant which alters the charge of the α-helix hinge to prevent it being repelled by the lobe, results in partially activated RNMT in the absence of RAM. The RNMT disulphide bridge mutants which prevent the α-helix hinge assuming favourable positioning, inhibit AdoMet binding and activity, and prevent RAM binding and stimulation of activity.

The positioning of the α-helix hinge contributes substantially to the stability of the active site by influencing the positioning of helix A, which contains three amino acids involved in hydrogen bonding with the substrates. Simulations indicate that one of these, K180, is critical for the relative positioning of substrates, allowing optimal in-line transfer of the methyl group. In simulations, helix A exhibits increased mobility in the absence of RAM. In the presence of RAM helix A is stabilised and constrained. The importance of the α-helix hinge in RNMT function is indicated in the hinge mutant, K409E K413E, which has reduced AdoMet binding and reduced activity. Furthermore, the hinge mutant has reduced interaction with and activation by RAM.

### Comparison with Vaccinia virus cap methyltransferase

**Figure 7.**
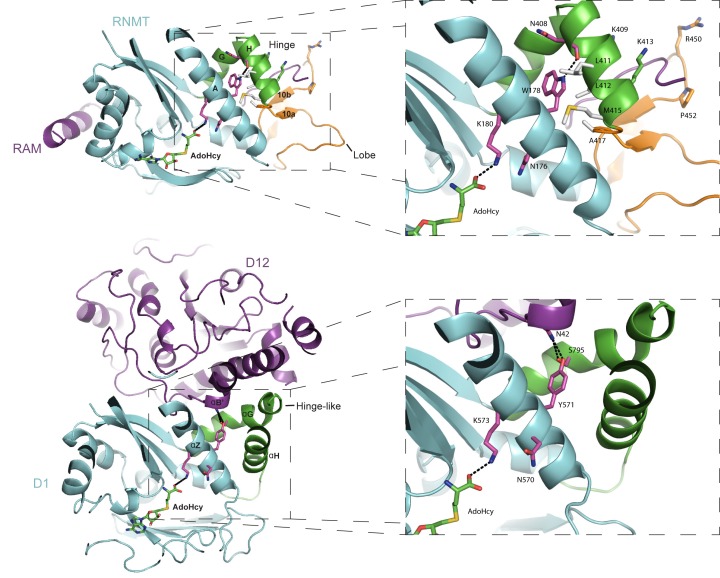
Comparision of RNMT–RAM structure with Vaccinia virus D1 cap methyltransferase and its activating subunit D12. RNMT and D1 are shown in cyan, whereas RAM and D12 in magenta. The RNMT hinge and the hinge-like region in D1 comprising of helices αG and αH are highlighted in green. D1 lacks a region with structural similarity to the RNMT lobe, which is shown in orange. The zoomed images demarcate active site residues within helix A and αZ of RNMT and D1 respectively. Residues involved in a polar interaction (dashed line) critical for the stability of helix A in RNMT and the corresponding residues in D1 have also been highlighted as sticks.

Human RNMT has 10–20% of the activity of RNMT-RAM, whereas Vaccinia virus cap methyltransferase has minimal activity in the absence of its activating subunit D12 (23,34,35). At first inspection the 33 kDa D12 and 14 kDa RAM have negligible sequence or motif similarity. The two proteins also show obvious structural differences when crystalised with the corresponding methyltransferases, D1 and RNMT (Figure 7). However, upon careful examination of the cap methyltransferase structures, parallel modes of action of RAM and D12 emerge. Similar to RAM, D12 binds to the cap methyltransferase surface distal to the active site and increases interaction with AdoMet and GTP/GpppA (16,25). Helix αZ of Vaccinia virus D1 contains two conserved active site residues, N570 and K573, and is braced by helices αG and αH over its N-terminus and helix αB’ of D12 over its C-terminus. D12 contacts helices αG and αH, thus stabilizing the entire length of helix αZ and causing the allosteric activation of D1. The alpha helix hinge and lobe of RNMT show sequence and positional similarities to helices αG and αH of D1. Helix A contains the corresponding active site residues in RNMT, N176 and K180, and is stabilized by RAM.

Individual alanine substitutions along the D1-D12 interface do not ablate the ability of D12 to bind or activate D1 (17). Similarly, we failed to detect alterations in activity following single or double amino acid substitutions in RAM (Supplementary Figure S5). However, di-alanine substitution N42 mutant of D12 was identified to be uniquely defective in D1 activation (36). This residue is involved in hydrogen bonding with S795 of αG and Y571 of αZ in D1. In our MD simulations of RNMT–RAM, we observe that the highly stable hydrogen bond between N408 of the hinge and W178 of helix A (distance of 3.1 Å ± 0.2) maintains W178 in a hydrophobic pocket formed by L411, L412, M415 and A417. Whereas the presence of RAM or activating mutation in RNMT stabilize this hydrogen bond (2.5 Å ± 0.2 in RNMT R450E P452E), mutations deleterious to RNMT activity were found to disrupt its formation (7.2 Å ± 1.4 in RNMT K409E K413E). RNMT W178 corresponds to Y571 in D1 and the position of N408 in the RNMT–RAM crystal structure is similar to that of N42 in D12. Thus in both human and vaccinia virus cap methyltransferases the hydrogen bonding between these two positions seems critical for the stability of helix A and methyltransferase activity.

Despite having evolved distinctly D12 and RAM binding to their cognate cap methyltransferase results in similar allosteric activation. RAM orthologs have only been identified in vertebrates, whereas D12 seems to be an isolated evolutionary acquisition. It is conceivable that these methyltransferase activators evolved to contend with increasing transcript complexity, to regulate mRNA cap methylation or as chance viral adaptation. Since these drastically different proteins exhibit functional similarity, it remains a possibility that in lower organisms unidentified peptides perform a similar role.

### Tuneable cap methylation

Here we present the molecular mechanism by which RAM increases RNMT cap methyltransferase activity. Comparison with the activation mechanism of Vaccinia virus cap methyltransferase suggests an evolutionary advantage of these allosteric activators. The ability of RAM to increase AdoMet recruitment to RNMT would confer a competitive advantage over the profusion of methyltransferases that function at sites of active transcription. In cellular systems, regulation of RAM expression would result in regulation of mRNA cap methylation and gene expression.

## Supplementary Material

SUPPLEMENTARY DATA
