# Supplementary material for: Molecular basis of RNA guanine-7 methyltransferase (RNMT) activation by RAM
Source: Nucleic Acids Res. 2016 Jul 15;44(21):10423–36. doi: 10.1093/nar/gkw637 (PMC5137418; doi:10.1093/nar/gkw637)
Supplement: SUPPLEMENTARY DATA [file supp_44_21_10423__index.html]

Molecular basis of RNA guanine-7 methyltransferase (RNMT) activation by RAM — SUPPLEMENTARY DATA 

# Molecular basis of RNA guanine-7 methyltransferase (RNMT) activation by RAM

## SUPPLEMENTARY DATA

- SUPPLEMENTARY DATA
- SUPPLEMENTARY DATA
- SUPPLEMENTARY DATA
