# Supplementary material for: Molecular basis of RNA guanine-7 methyltransferase (RNMT) activation by RAM
Source: Nucleic Acids Res. 2016 Jul 15;44(21):10423–36. doi: 10.1093/nar/gkw637 (PMC5137418; doi:10.1093/nar/gkw637)
Supplement: SUPPLEMENTARY DATA [file supp_gkw637_nar-01190-z-2016-File003.pdf]

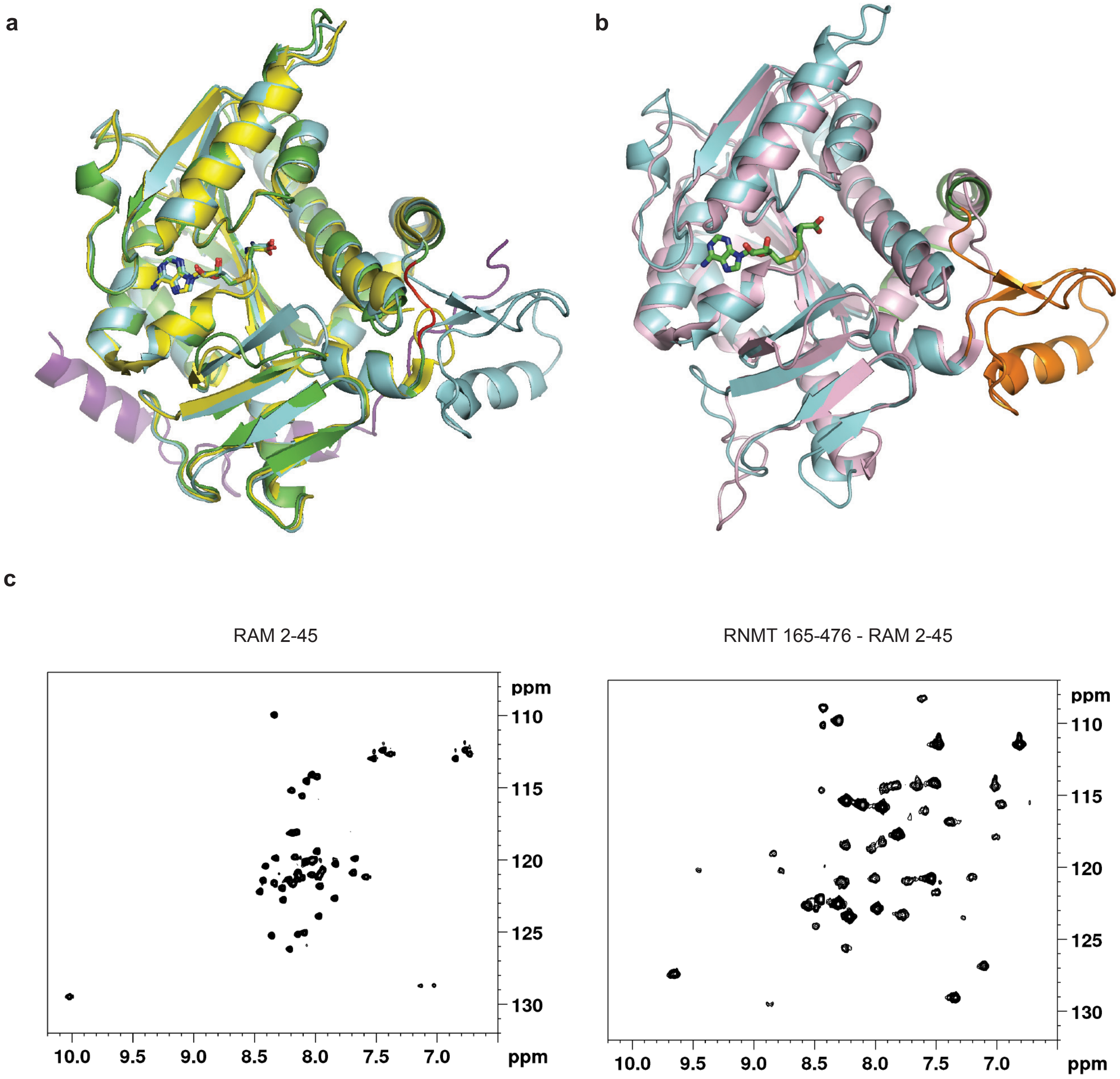

Supplementary Figure 1: RNMT - RAM interaction does not alter the RNMT core structure but stabilises RAM.
a) Superposed crystal structures of RNMT 165-476 lobe deletion mutant (Δ416-456) in green containing GSGG linker shown in red, RNMT 165-476 following limited proteolysis with thermolysin shown in yellow and RNMT 165-476 - RAM 2-45 shown in cyan and purple respectively (RMSD ~ 0.5Å). b) Superposition of RNMT 165-476 from the RNMT-RAM structure in cyan with Ecm1 in pink (PDB:1RI3). c) HSQC NMR spectra of 15N-labelled RAM 2-45 in absence or presence of unlabelled RNMT 165-476.

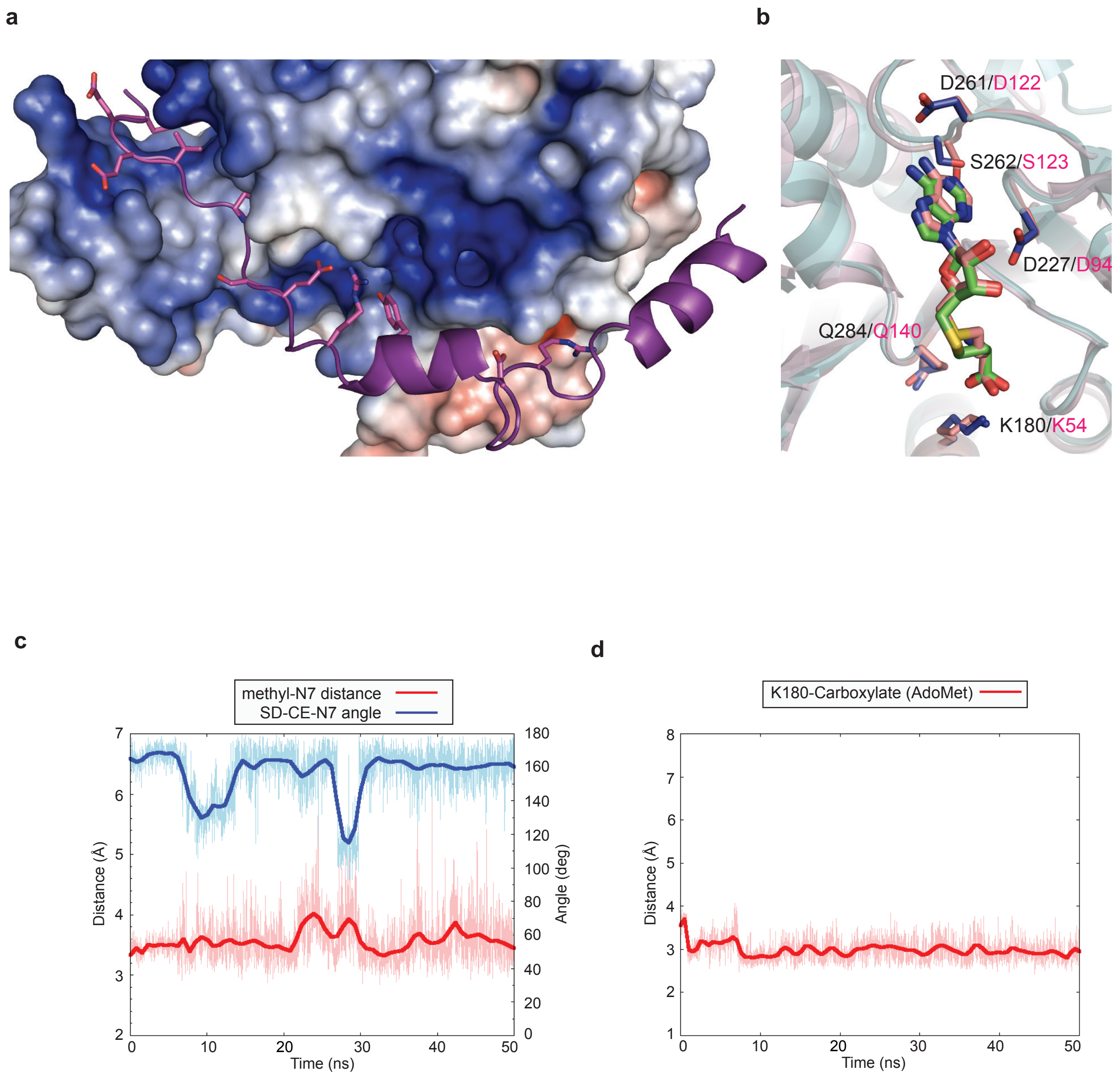

Supplementary Figure 2: RAM binds surface grove on RNMT but does not alter the active site. a) RNMT electrostatic potential plotted on the surface as calculated by APBS from the crystal structure (-5/+5 KbT/e). Positive charge is depicted as blue and negative charge as red. b) Alignment of residues involved in AdoMet binding for RNMT 165-476 - RAM 2-45 in cyan and Ecm1 in pink (PDB: 1RI3). For simplicity, only residues involved in polar interactions are highlighted as sticks, residues in black correspond to RNMT-RAM and in pink correspond to Ecm1. c) Time evolution of the methyl-N7 distance and SD-CE-N7 angle during a 50ns MD simulation. d) Time evolution of the K180 interaction with AdoMet carboxylate groups during a 50ns MD simulation.

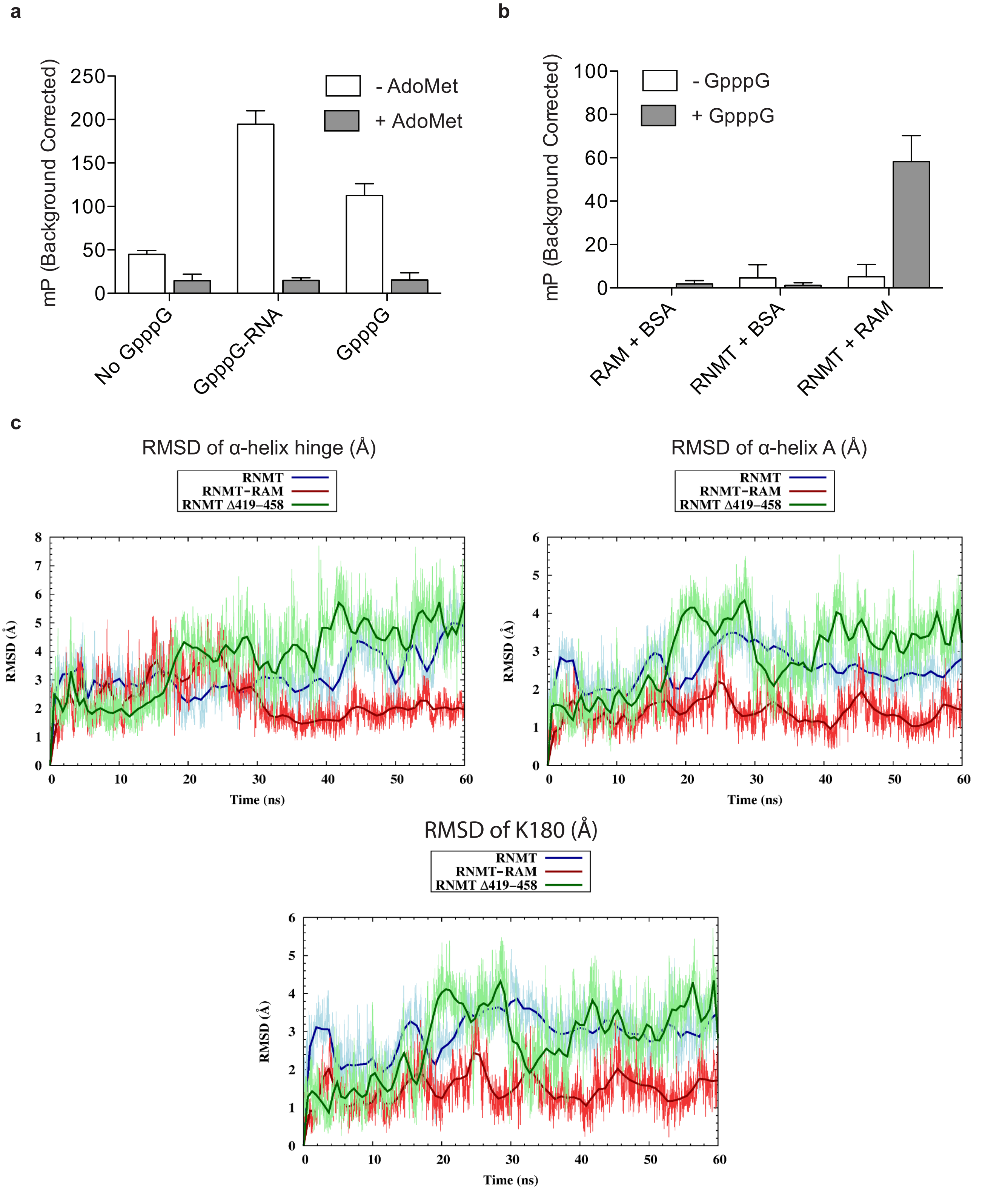

Supplementary Figure 3: RAM and cap increase AdoMet recruitment by RNMT. a) Maximal polarisation for fluorescent SAM probe (Cayman Chemical) binding to RNMT-RAM 2-45 complex in the absence or presence of GpppG-RNA or GpppG analogue. Readings in the presence of AdoMet provide the specificity control. b) Maximal polarisation for SAM probe binding to RAM 2-45, RNMT or RNMT - RAM 2-45 complex in the absence or presence of GpppG analogue. All values represent mean ± S.D. in all panels (n = 3). c) RMSD of α-helix A or α-helix hinge or K180 as calculated from the 60ns MD trajectories of the RNMT monomer, RNMT-RAM complex or RNMT Δ416-456.

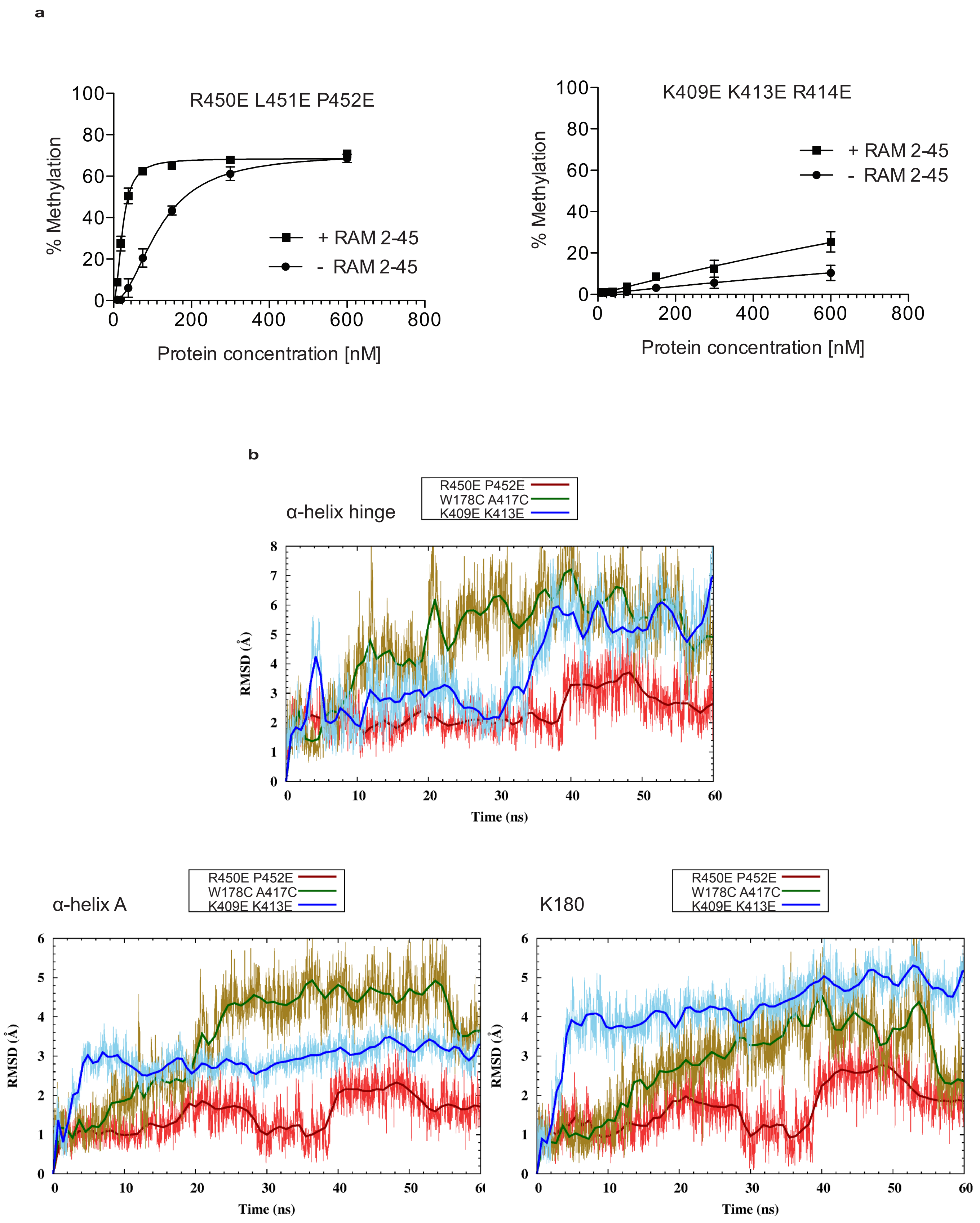

Supplementary Figure 4: Charge alterations in the RAM binding groove affect RNMT enzymatic activity. a) Methyltransferase activity assays performed with RNMT R450E L451E P452E and RNMT K409E K413E R414E in the absence or presence of RAM 2-45. Values represent mean ± S.D (n=3). b) RMSD of helix A, hinge and K180 as calculated from the 60ns high-temperature MD trajectories of the RNMT R450E P452E, RNMT K409E K413E and RNMT W178C A417C.

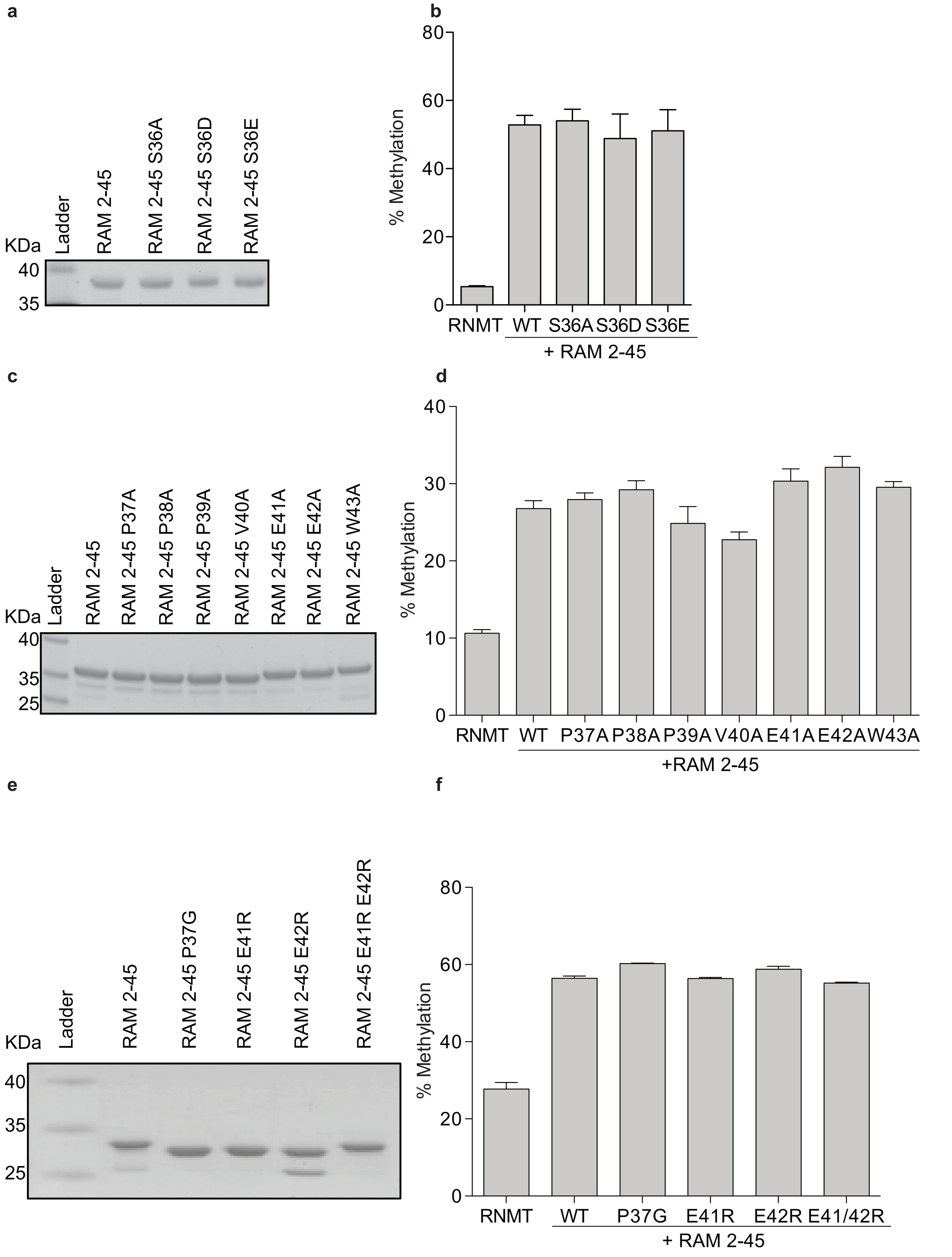

Supplementary Figure 5: RAM mutant analysis. a,c,e) Coomassie stained SDS-PAGE gel analysis of purified recombinant RAM mutants. b,d,f) Methyltransferase activity assays performed with 100nM RNMT and 100nM RAM 2-45 (WT) or the specified mutant. Values depict mean ± S.D. for three technical replicates.

Supplementary Table 1: Polar interactions between RNMT and RAM in the crystal structure and MD simulations. The analysis performed for the 2 complexes of the asymmetric unit using the PISA server (EMBLI-EBI). Only data for chains B and D are shown. All the main interactions are conserved.

RAM		RNMT		Distance (Å) in crystal structure	Distance (Å) in MD simulations
ARG	18 [NH1]	TYR	351 [O]	2.64	2.7 (± 0.1)
ASP	23 [OD2]	TYR	374 [OH]	3.26	4.5 (± 0.9)
TYR	29 [OH]	GLU	323 [OE2]	2.43	2.8 (± 0.3)
GLU	25 [OE1]	LYS	392 [NZ]	3.86	5.2 (± 1.8)
TYR	29 [OH]	ASN	320 [ND2]	3.22	3.8 (± 0.6)
ARG	32 [NH2]	GLU	397 [OE1]	2.71	4.5 (± 1.8)
GLU	35 [OE1]	TRP	461 [NE1]	3.59	3.2 (± 0.5)
VAL	40 [N]	GLY	454 [O]	2.79	3.0 (±0.1)
VAL	40 [O]	GLY	454 [N]	2.58	2.9 (± 0.2)
GLU	41 [OE1]	TYR	443 [OH]	2.36	3.3 (± 0.8)
GLU	41 [OE2]	ARG	450 [N]	3.31	3.7 (± 1.0)
GLU	42 [N]	ARG	450 [O]	2.85	3.1 (± 0.3)
GLU	42 [N]	PRO	452 [O]	3.19	3.2 (± 0.3)
GLU	42 [O]	LYS	413 [NZ]	3.21	3.5 (± 1.2)
GLU	42 [OE1]	ARG	450 [NH1]	3.13	4.9 (± 1.6)
TRP	43 [N]	PRO	452 [O]	2.99	2.9 (± 0.2)

Supplementary Table 2. Summary of molecular dynamics simulations performed in this work.

MD Simulation System	Temperature	
	300 K	400 K (2 replicas)
RNMT	400 ns	60ns
RNMT + RAM	400 ns	60ns
RNMT + RAM + ligands (AdoMet and Gppp)	50ns	-
RNMT Δ419-458	400 ns	60ns
RNMT R450/P452E	400 ns	60ns
RNMT W178/A417C	400 ns	60ns
RNMT K409/K413E	400 ns	60ns

Supplementary Methods for Varshney and Petit et al,

Recombinant protein purification for X-ray crystallography

The human RNMT catalytic domain (Uniprot ID:O43148 - fragment 165-476) was expressed in the E.coli BL21 Codon Plus RIL strain as a TEV-cleavable His tag fusion. RNMT Δ416-456 (fragment 165-476) was expressed in the E.coli BL21 gold strain as a TEV-cleavable MBP-His fusion. RAM (fragment 2 to 45) was expressed in the E.coli BL21 gold strain as a prescission-cleavable GST fusion. Both strains were grown in ZY auto-induction medium at 17 degrees overnight. The procedure for the purification of RNMT was updated from Lunin et al, 2007 (PDB: 3BGV). The RNMT catalytic domain (wild type and Δ416-456) was initially purified by metal affinity chromatography followed by a buffer exchange step to remove the imidazole. The desalted fractions were incubated with the TEV protease (1/20 m/m) overnight at 4 degrees prior to a second purification by metal affinity chromatography. The flow through was further purified by gel permeation. Monomeric fractions were pooled and diluted 6 times in Pipes 50mM, 10% glycerol pH 6.5 and purified by cation exchange chromatography. RAM 2-45 was purified using glutathione sepharose high performance beads. A desalting step was added to remove glutathione prior to the cleavage using the prescission protease (1/20 m/m) overnight at 4 degrees. RAM was then purified by gel filtration chromatography.

The RNMT-RAM complex was obtained by incubating the purified RNMT and RAM proteins (molar ratio 1:1.2) in 20 mM Pipes (pH 6.5), 150 mM NaCl, 50mM KCl, 1 mM TCEP [Tris(2-carboxyethyl)phosphine hydrochloride] and 5% glycerol at 4°C for 2 h. The complex was separated from the excess free RAM by gel filtration chromatography and concentrated to 34mg/ml in presence of 5mM SAH.

X-ray diffraction data collection

The RNMT-RAM X-ray diffraction data were collected at 100 K at beamline ID24 - (Diamond synchrotron). The data were processed using XDS (37) and scaled using SCALA

(38) from the CCP4 suite (39). The crystal diffracts to 2.35 Å and belongs to the space group P1. The cut-off criteria used were I/σ(I)>2.0 and Rmerge<50% for the outer shell as the CC(1/2) was not available due to the low redundancy of the collected data. The initial phase information was obtained by molecular replacement using Phaser from the CCP4 suite and the human catalytic domain RNMT as model (PDB ID: 3BGV). The initial densities were further improved by solvent flattening and histogram matching using RESOLVE (40), as implemented in the Phenix suite (41). The RAM molecule was built manually by iterative cycles of model building and refinement using COOT (42) and Refine (Phenix suite).

Diffraction data from the RNMT lobe deletion mutant and the thermolysin-cleaved RNMT were collected at 100K at beamlines ID30 (ESRF synchrotron) and ID04 (Diamond synchrotron) respectively. The structure determination method previously described for RNMT-RAM was used to solve and refine the structures of the RNMT lobe deletion / thermolysin structures. The crystals diffract to respectively 3.47 Å and 2.28 Å and belong to the space group $P2_12_12_1$ with CC(1/2) for the outer shell of 55% and 95% respectively.

Recombinant protein purification for biochemical assays

Variations of His-RNMT FL or GST-RAM 2-45 were expressed in BL21 *E. coli* plus RIL from pET156P or pGEX6P1 vectors, respectively. Bacteria were cultured in Terrific Broth at 37°C to an A_{600} of 1.2 and protein expression induced with 0.4mM IPTG overnight at 16°C. Cells were harvested by centrifugation, washed in ice-cold PBS and lysed in 50mM Tris-Cl pH7.5, 250mM NaCl, 0.5mM EDTA, 0.5mM EGTA, 0.5% Triton-X100, 1mM DTT, 20ug/Ml Leupeptin, 1mM Pefabloc, 0.5mM TCEP by sonication. Glutathione or Nickel sepharose was incubated with the soluble fraction for an hour at 4°C and washed twice with 50mM Tris-CL pH7.5, 250mM NaCl, 0.03% Brij-35, 1mM DTT. His-RNMT FL was eluted in 50mM Hepes pH7.5, 270 mM sucrose, 150mM NaCl, 0.03% Brij-35, 1mM DTT, 400mM Imidazole and RAM 2-45 in 50 mM Tris-Cl pH 7.5, 270 mM sucrose, 150 mM NaCl, 0.1 mM EGTA, 0.1 % β-mercaptoethanol, 0.03% Brij-35, 10mM L-Glutathione. GST tag was cleaved

by incubation with GST-TEV protease at 4°C overnight and cleaved protein purified by passing through a glutathione sepharose column. All proteins were purified by size exclusion chromatography on a SD200 column (GE Healthcare), quantified by A_{280} with a Nanodrop and analysed by SDS-PAGE prior to use. RNMT disulphide bridge mutants were purified in the absence of reducing agents.

Mass Spectrometry

2-3 mg of each ligand was dissolved in 1mL of DMSO (required sonication). 5µL of each solution was dissolved in 1mL H_2O to give approx. 20µg/mL solution. Each standard was run with either basic (0.1% NH_3) or acidic (0.1% formic acid) eluents on a Waters BEH C18 column. As the ligands are particularly polar a number of gradients were tried. 100uL of the protein solution was precipitated by addition of 300µL ACN or addition of 5µL TFA. Protein samples were vortexed, centrifuged and the supernatant taken and added to an equal volume of H_2O.

LC/MS data was generated using a Waters Xevo Q-TOF quadrupole time-of-flight hybrid mass spectrometer in positive ion electrospray ionization (ESI). The inlet system consisted of a Waters Acquity UPLCTM system with a diode-array detector. Data were acquired from 50 to 1000 Da with a 0.2 s scan time, using a desolvation temperature of 500°C, source temperature of 120°C, and cone voltage of 15 V. The mass spectrometer was calibrated across the mass range 50–1000 Da using a solution of sodium formate. Data were centroided and mass corrected during acquisition using an external reference (Lock-SprayTM) comprising a 0.2 ng/µL solution of leucine encephalin infused at 50µL/min, generating a reference ion at 556.2771. 2µL was injected onto a Waters Acquity BEH C18 column (50x2.1mm, particle size 1.7mm) held at 40C. Eluents used were A – 0.1% formic in H2O (acidic) or 0.1% NH3 in H2O (basic), B – 0.1% formic in ACN (acidic) or 0.1% NH3 in ACN (basic). The gradient was held at 100% A for 0.5min, then a linear gradient to 65% A

after 4 min, then to 5% A after 5 min, held for 1 min before returning to 100% A. The flow rate was 0.5mL/min.

Cell culture

HeLa cells were cultured in DMEM/10% FBS at 37°C in 5% CO_2. Transient transfections were performed using Lipofectamine® 2000 (Invitrogen) with 4μg pcDNA5, 2μg pcDNA5-HA-RNMT and 4μg pcDNA5-HA-RNMT Δ416 - 456 for 48 hours, to balance expression.

Molecular dynamics (MD) simulations

1. Simulation system setup

Using the crystal structure of RNMT-RAM (PDB: 5E8J) six different simulation systems were constructed: 1) RNMT, 2) RNMT-RAM, 3) RNMT Δ419-458, 4) RNMT R450E P452E, 5) RNMT W178C A417C, and 6) RNMT K409E K413E. All models were prepared with the LEaP utility from the AMBER14 suite of programs (27) using the ff14SB force field (28). The N-terminus of RNMT was capped with an acetyl group (ACE), and the N- and C-termini of RAM were capped with ACE and a methylated amino group (NME), respectively.

In addition, for the RNMT-RAM complex, a simulation system with ligands - Gppp and AdoMet - was built. Gppp was introduced to RNMT by aligning the protein with a Gppp-bound structure of the *E. cuniculi* mRNA cap methyltransferase, Ecm1 (15) (PDB: 1RI2) and superposing Gppp to the RNMT active site. The force field parameters for Gppp and AdoMet were generated using the Antechamber utility in combination with the general AMBER force field (GAFF)(43). Atomic charges were assigned using the AM1-BCC method (44,45).

Each simulation system was immersed in a cubic box of TIP3P water molecules that extended at least 15 Å away from protein, and then was neutralized by adding the appropriate number of Cl^- or Na^+ ions (46).

2. MD details and simulation protocol

All MD simulations were performed using the *pmemd* module of AMBER14. Periodic boundary conditions were used. The cut-off distance for the non-bonded interactions was 12 Å. Electrostatic interactions were treated using the smooth particle mesh Ewald method. The SHAKE algorithm was applied to all bonds involving hydrogens, and an integration step of 2.0 fs was used throughout (47) . Each system was first minimized (3,000 steps of steepest-descent energy minimization) to remove steric clashes, and then heated to 300/400 K over a 50 ps interval using a weak coupling algorithm (48). A 200 ps equilibration was performed to allow the solvent to redistribute around the positionally restrained protein. The system was subsequently allowed to evolve freely unrestrained at constant temperature (300 K or 400 K) and pressure (1 atm) using the weak-coupling algorithm (48). Atomic coordinates were collected every 20 ps for further analysis.

The activation mechanism of RNMT involves protein-protein binding and large-scale conformational changes. It is a challenging task for standard atomistic simulations to properly sample such processes because of their long timescales and/or high conformational energy barriers. Therefore, along with MD simulations at a 'standard' temperature (300 K), high-temperature calculations (at 400 K) were performed to induce larger conformational changes in RNMT within reasonable simulation time. A total of 13 MD simulations were carried out: six systems (RNMT, RNMT-RAM, RNMT Δ419-458, RNMT R450E P452E, RNMT W178C A417C, and RNMT K409E K413E) were simulated at both 'standard' (300 K) and 'high' (400 K) temperature for 400 ns and 60 ns, respectively (Table S2). In addition, a 50 ns MD simulation (at 300 K) was carried out for the RNMT-RAM system with the ligands (AdoMet and Gppp) bound in the active site.

3. Analysis of MD trajectories

Protein structures and MD trajectories were visually inspected using the molecular visualization programs PyMOL (49) and VMD (50). Interatomic distances and angles, as well as root-mean-square deviations (rmsd) with respect to a reference structure, were monitored using the *cpptraj* module in AmberTools 15 (27(51). The heatmap plots were created using the *gnuplot* program.

MD simulation (First nucleotide binding).

m7Gppp was introduced to the RNMT structure as described above. The phosphate chain was methylated to neutralize the additional charge without significantly restricting the freedom of movement during the optimization of the binding mod-e. Restraints were set between the N7-Me and AdoHcy sulphur (160˚-180 ˚, S – N7: 3.6Å-3.9Å, S – C: 1.8 Å – 2.2 Å) with weight 30 in MOE (52). The structure was minimized (Amber12:EHT) in a water box with progressively weakening heavy atom restraints and relaxed further in a short 1.2 ns MD simulation. The first nucleotide guanine was added to m7Gppp using a restrained docking in Glide (53) and m7GpppG binding was optimized once more as described above.

References:

37. Kabsch, W. (2010) Xds. *Acta crystallographica. Section D, Biological crystallography*, **66**, 125-132.
38. Evans, P. (2006) Scaling and assessment of data quality. *Acta crystallographica. Section D, Biological crystallography*, **62**, 72-82.
39. Collaborative Computational Project, N. (1994) The CCP4 suite: programs for protein crystallography. *Acta crystallographica. Section D, Biological crystallography*, **50**, 760-763.
40. Terwilliger, T.C., Grosse-Kunstleve, R.W., Afonine, P.V., Moriarty, N.W., Zwart, P.H., Hung, L.W., Read, R.J. and Adams, P.D. (2008) Iterative model building, structure refinement and density modification with the PHENIX AutoBuild wizard. *Acta crystallographica. Section D, Biological crystallography*, **64**, 61-69.
41. Adams, P.D., Afonine, P.V., Bunkoczi, G., Chen, V.B., Davis, I.W., Echols, N., Headd, J.J., Hung, L.W., Kapral, G.J., Grosse-Kunstleve, R.W. *et al.* (2010) PHENIX: a comprehensive Python-based system for macromolecular structure solution. *Acta crystallographica. Section D, Biological crystallography*, **66**, 213-221.
42. Emsley, P. and Cowtan, K. (2004) Coot: model-building tools for molecular graphics. *Acta crystallographica. Section D, Biological crystallography*, **60**, 2126-2132.
43. Wang, J., Wolf, R.M., Caldwell, J.W., Kollman, P.A. and Case, D.A. (2004) Development and testing of a general amber force field. *Journal of computational chemistry*, **25**, 1157-1174.
44. Jakalian, A., Bush, B.L. and Jack, D.B. (2000) Fast, efficient generation of high-quality atomic charges. AM1-BCC model: I. Method. *Journal of computational chemistry*, **21**, 132-146.
45. Jakalian, A., Jack, D.B. and Bayly, C.I. (2002) Fast, efficient generation of high-quality atomic charges. AM1-BCC model: II. Parameterization and validation. *Journal of computational chemistry*, **23**, 1623–1641.
46. Åqvist, J. (1990) Ion-water interaction potentials derived from free energy perturbation simulations. *The Journal of Physical Chemistry*, **94**, 8021-8024.
47. Ryckaert, J.-P., Ciccotti, G. and Berendsen, H.J.C. (1977) Numerical integration of the cartesian equations of motion of a system with constraints: molecular dynamics of n-alkanes. *Journal of Computational Physics*, **23**, 327-341.
48. Berendsen, H.J.C., Postma, J.P.M., van Gunsteren, W.F., DiNola, A. and Haak, J.R. (1984) Molecular dynamics with coupling to an external bath. *The Journal of Chemical Physics*, **81**, 3684–3690.
49. Delano, W.L. (2002) The PyMOL Molecular Graphics System. *Schrödinger.*
50. Humphrey, W., Dalke, A. and Schulten, K. (1996) VMD: visual molecular dynamics. *Journal of molecular graphics*, **14**, 33-38, 27-38.
51. Roe, D.R. and Cheatham, T.E.I. (2013) PTRAJ and CPPTRAJ: Software for Processing and Analysis of Molecular Dynamics Trajectory Data. *Journal of Chemical Theory and Computation*, **9**, 3084-3095.
52. MOE. (2015). 1010 Sherbooke St. West, Suite #910, Montreal, QC, Canada, H3A 2R7.
53. Glide. (2015). Schrödinger Inc.
